# Regulatory B Cells Dysregulated T Cell Function in an IL-35-Dependent Way in Patients With Chronic Hepatitis B

**DOI:** 10.3389/fimmu.2021.653198

**Published:** 2021-04-12

**Authors:** YaYun Liu, Ying Luo, Tong Zhu, Meng Jiang, ZhaoFeng Tian, GuSheng Tang, XueSong Liang

**Affiliations:** ^1^ Department of Infectious Diseases, Changhai Hospital, Navy Military Medical University, Shanghai, China; ^2^ Department of Clinical Experiment Center, Changhai Hospital, Navy Military Medical University, Shanghai, China; ^3^ Department of Laboratory Diagnostics, Changhai Hospital, Navy Military Medical University, Shanghai, China; ^4^ Department of Hematology Laboratory Center, Changhai Hospital, Navy Military Medical University, Shanghai, China

**Keywords:** chronic HBV infection, regulatory B cells (Bregs), interleukin 35 (IL-35), IL-35-secreting B (IL-35+B) cells, HBV - hepatitis B virus, immune regulation

## Abstract

Interleukin (IL)-35-secreting B (IL-35+B) cells are critical regulators in autoimmune and infectious diseases and exert suppressive functions in parallel with IL-10-producing B (B10) cells. However, the role of IL-35+B cells in persistent hepatitis B virus (HBV) infection remains unclear. To elucidate the role of IL-35+B cells in the progress of chronic HBV infection, we determined the frequency of IL-35+B cells and their relationship with the classical human regulatory B cell (Breg) subsets, namely, CD19+CD24^hi^CD38^hi^ and CD19+CD24^hi^CD27+. Then, the regulatory effect and mechanism of Bregs on effector T cells were investigated *in vitro*. Here, we found that compared with healthy controls, the frequency of IL-35+B cells was increased in patients with chronic HBV infection and was enriched in human classical Breg subset CD19+CD24^hi^CD38^hi^ B cells. Moderate correlation was observed between the frequency of IL-35+B cells and alanine aminotransferase levels (Spearman r = 0.401), but only mild correlation was noted between the frequency of IL-35+B cells and HBV DNA level (Spearman r = 0.314). The frequency of IL-35+B cells was negatively correlated with interferon-γ (IFN-γ)-producing CD4+ and CD8+ cells but positively correlated with IL-4-producing T cells. Bregs dysregulated T cell function through an IL-35-dependent mechanism and depended on cell-to-cell contact. In conclusion, IL-35+ B cell was enriched in CD19+CD24hiCD38hi B cell subset during persistent HBV infection and Breg cells exerted dysregulation in T cell function through IL-35 dependent mechanism and depend on cell-to-cell contact.

**Clinical Trial Registration:**

www.ClinicalTrials.gov, identifier NCT03734783.

## Introduction

Chronic hepatitis B virus (HBV) infection is a major global health concern that is correlated with the occurrence of liver cirrhosis and hepatocellular carcinoma and affects more than 200 million patients worldwide (1). Host acquired antiviral immunity, especially T cell immunity, is vital in HBV clearance during acute and chronic HBV infection, however, once a chronic HBV infection is established, viral-specific T cell immunity is manifested as functional imbalance or exhaustion, and the mechanism is not yet fully understood (2–6). Immune suppressor cells such as CD4+CD25^high^FoxP3+ regulatory T cells (Tregs) (4), costimulatory factors such programmed death factor 1 pathway (7, 8), and Treg/Th17 imbalance (9, 10) are important in the exhaustion of T cell immune function during chronic HBV infection.

Regulatory B cells (Bregs), newly identified regulatory cells, exert immunoregulatory roles in the course of infectious diseases (11, 12), autoimmune diseases (13, 14), and cancer (15, 16) by secreting interleukin (IL)-10, transforming growth factor-β1 (TGF-β1), IL-35, and other inhibitory cytokines. Das et al. (17) studied the role of Bregs in persistent HBV infection and found that IL-10-producing B (B10) cells are enriched in patients with chronic hepatitis B (CHB) and the frequency of B10 cells is correlated temporally with hepatic flares. In another study, Gong et al. (18) simply defined Bregs as CD19+IL-10+ cells and demonstrated that these Bregs have similar immune regulatory function to Tregs during chronic HBV infection.

IL-35 (p35/Ebi3), an anti-inflammatory cytokine of the IL-12 family, which is highly expressed in CHB patients and plays an important role in the inhibition of cellular immune response (19–21), is another major effector cytokine of Bregs. Shen et al. (22) had demonstrated that IL-35-secreting B (IL-35+B) cells are another critical regulator during autoimmune and infectious diseases and exert suppressive functions in parallel with B10 cells. However, the role of IL-35+B cells in CHB remains unclear. In the present study, we aimed (1) to examine the frequency of IL-35+B cells in total peripheral blood B cells of CHB patients, (2) to determine the frequency of IL-35+B cells in two humans classical Breg subsets in CHB, (3) to investigate whether Bregs regulated T cell function through IL-35 pathway, and (4) to explore the possible mechanism of Bregs on effector T cells.

## Materials and Methods

### Patients and Controls

All individuals were recruited according to the protocol that was approved by the clinical Ethics Committees of Shanghai Changhai Hospital (CHEC2017-118) and registered on *Clinicaltrials.gov* (NCT03734783). The study was conducted between July 2017 and November 2019. Written informed consent was obtained from all participants.

Patients with CHB were all sero-positive for hepatitis B surface antigen (HBsAg) for at least 6 months and negative for other hepatotropic viruses, such as hepatitis D virus (HDV), hepatitis C virus (HCV), hepatitis E virus, hepatitis A virus, and human immunodeficiency virus 1/2. Healthy controls (HCs) did not have any previous history and current evidence of liver disease. Serum alanine aminotransferase (ALT) values were normal, and HBsAg and other hepatitis virus markers were negative. Furthermore, all participants were sero-negative for markers such as ceruloplasmin, anti-nuclear antibodies and anti-mitochondrial antibodies for co-existent autoimmune and metabolic liver diseases.

### Direct Labeling and Intracellular Labeling Flow Cytometric Analysis

Peripheral blood mononuclear cells (PBMCs) were isolated using lymphocyte separation medium (Ficoll-Hypaque density gradient, Axis-shield, Germany). The antibodies and fluorochromes used in this work are shown in **Table S1**.

For Breg subset and B10 or IL-35+B cell detection, PBMCs (2 × 10^6^) were stimulated with CD40L (5 µg/mL, Purified Anti-Human CD154 (CD40L), Tonbo Biosciences, USA) plus CpG-ODN (1.5 µM, TLR9 Agonist-Stimulatory Class B tlrl-2006, InvivoGen) and lipopolysaccharide (LPS, 1 µg/mL, eBioscience) for 48 h. Furthermore, cells were activated for another 4 h at 37°C in 5% CO_2_ with 50 ng/mL phorbol myristate acetate, 1 mmol/L ionomycin (both from Sigma, St. Louis, MO, USA), and 10 mg/mL brefeldin A (Tocris Cookson, Bristol, UK) in complete RPMI-1640 (Invitrogen, Carlsbad, CA, USA) supplemented with 10% heat-inactivated fetal bovine serum (Gibco, Grand Island, NY, USA). The cells were then collected and washed with PBS once and stained for surface markers. Then, they were permeabilized with Perm/Fix solution (Cytofix/Cytoperm™, BD biosciences) and stained intracellularly with specific fluorescent conjugated anti-human IL-10, eBi3/IL-12/IL-35p35 (IL-35). For B cell subsets, we analyzed two classical Breg subsets: CD24^hi^CD38^hi^, CD24^hi^CD27+, the total IL-10 secreting B cells and IL-35 secreting B cells, which were all gated on CD19+ cells (**Figure S1B**). We also determined the frequency of IL-35 producing Breg: CD24^hi^CD38^hi^IL-35+, CD24^hi^CD27+IL-35+, and IL-10 secreting Breg: CD24^hi^CD38^hi^IL-10+, CD24^hi^CD27+IL-10+, frequency was determined into CD24^hi^CD38^hi^ and CD24^hi^CD27+ gates, respectively (**Figure S1**).

For T cell subsets detection, freshly isolated PBMCs (2 × 10^6^) were stimulated for 5 h with 50 ng/mL phorbol myristate acetate,1 mmol/L ionomycin (both from Sigma, St. Louis, MO, USA), and 10 mg/mL brefeldin A (Tocris Cookson, Bristol, UK) in complete RPMI-1640 (Invitrogen, Carlsbad, CA, USA) supplemented with 10% heat-inactivated fetal bovine serum (Gibco, Grand Island, NY, USA). Upon harvest, cells were first stained with surface markers (CD3-APC-Cy7-A, CD4-FITC-A, and CD8-PerCP-Cy5-5-A) and then intracellularly stained with IL-17-APC-A, IFN-γPE-Cy7-A, and IL-4-PE-A. All these T cell subsets were gated on PBMC cells (**Figure S2**).

Flow cytometry was performed using a FACSCalibur (Becton Dickinson, San Jose, CA). FACS data were analyzed using CellQuest software (Becton Dickinson Rutherford, NJ).

### Luminex Multiplex Cytokine Assays

Serum concentrations of inflammatory cytokines, including IL-4, IL-17A, IL-21, and interferon-γ (IFN-γ), were measured using commercially available Luminex MAP kits (ProcartaPlex 5 plex, 1 plate 5-plex, eBioscience) in accordance with the manufacturer’s instructions. Samples were two times diluted with 1×universal assay buffer and tested in triplicate.

### Enzyme-Linked Immunosorbent Assay

Serum and PBMC culture supernatant concentrations of IL-35 and IL-10 were measured by using commercially available ELISA kits (XpressBio, USA; Proteintech, USA) in accordance with the manufacturers’ instructions. All samples were not diluted and assessed in triplicate.

### Suppression Effect on T Cells

Purified B cells isolated from PBMC using anti-human CD19 microbeads (Miltenyi Biotec, Bergisch Gladbach, Germany) were treated with CpG/CD40L/LPS with or without HBVcore (1-183 a.a) (ProSpec, USA) for 48 h. After stimulation, B cells were washed with PBS twice and then co-cultured with autologous CD19-depleted PBMCs (50,000 cells) at the ratio of 2:1 in anti-CD3/CD28 (working concentration of anti-CD3 and anti-CD28 was 10μg/ml and 2μg/ml respectively, Tonbo Biosciences, San Diego, CA)-coated 96-well plate in complete RPMI-1640 (Invitrogen, Carlsbad, CA, United States) supplemented with 10% heat-inactivated fetal bovine serum (Gibco,Grand Island, NY, United States) for another 48 h. After 48 h of co-culture, the frequency of IL-4-, IFN-γ-, and IL-17A-secreting T cells was evaluated by flow cytometry.

### Transwell Assay and Blocking Study

Transwells of 0.4 µm pore size were used (Millicell, Merck Millipore, Billerica, MA, USA). CD19-depleted PBMCs (50,000 cells) were added to the lower chambers of 24-well plates and subsequently stimulated with anti-CD3/anti-CD28 as described above, whereas non-treated or stimulated B cells (100,000) were added to the upper chambers in the transwells. As controls for the transwell assay, CD19-depleted PBMCs (50,000 cells) and stimulated B cells (100,000 cells) were also co-cultured in the same plate in the lower chambers with anti-CD3/anti-CD28. After 48 h of co-culture, CD19-depleted PBMCs were gathered, and the frequency of IFN-γ-secreting T cells was tested by flow cytometry. For cytokine blocking study, anti-IL-12/IL-35 p35 (1 µg/mL, R&D Systems) and anti-IL-10 (5 µg/mL, Invitrogen) were added to the co-culture system to investigate the potential mechanism of Breg on T cells.

### HBV DNA Quantification

Serum HBV DNA levels were quantified using fluorescent quantitative PCR with commercially available kits (Sansure Biotech, China). The detection range was 1 × 10^2^ to 5 × 10^8^ IU/mL.

### Viral Serological Test

The levels of HBsAg, HBeAg, anti-HBs, anti-HBc, anti-HBe, anti-HCV, anti-HDV, anti-HGV, anti-HIV-1, and anti-HIV-2 were measured using commercially available kits (Abbott Laboratories, North Chicago, IL) in our clinical laboratory. The dynamic range of serum HBsAg was 0.05–250 IU/mL. The samples were diluted to 1:500 or 1:1000 using the ARCHITECT HBsAg Manual Diluent (Abbott Diagnostics) if >250 IU/mL.

### Statistical Analysis

Normally distributed continuous quantitative data were expressed as the mean ± SEM, and non-normally distributed continuous quantitative data were expressed as median (interquartile range,IQR). The non-parametric Mann–Whitney U test was used to evaluate the differences between two independent samples, and the non-parametric Kruskal–Wallis ANOVA test was used to evaluate the differences in more than two groups. The non-parametric Friedman ANOVA test was used to evaluate effector T cell proportions in co-culture experiments. The statistical correlation between variables was calculated by Spearman rank correlation analysis. P-value < 0.05 was considered statistically significant. All analyses were performed using SPSS software (version 21.0.0; Chicago, IL). The graph was made using GraphPad Prism 5 (San Diego, CA, USA).

## Results

### Patients’ Clinical Characteristics

The clinical and biochemical characteristics of the studied patients are listed in **Table 1**. All 68 CHB patients were treatment-naïve at enrollment. Approximately 57.35% (39/68) of patients had ALT levels greater than the upper limit of normal (ULN) at enrollment, and higher proportions of patients in the abnormal ALT group were HBeAg positive compared with those in the normal ALT group. Similarly, HBeAg-positive patients at enrollment had higher median ALT levels than HBeAg-negative patients. Twenty-six age- and gender-matched HCs were enrolled.

**Table 1:** Individuals demographic and clinical characteristics.

Characteristics	HCs	CHB	ALT nor.	ALT abnor.	HBeAg pos.	HBeAg neg.	P* value	P^#^ value	P^$^ value
**Number**	26	68	29	39	39	29	/	/	
**Age (years)**	33.24 ± 8.99	36.84 ± 8.67	34.00(29.50,42.50)	35.00(29.00,41.75)	34.00(28.75,41.25)	36.00(31.00,43.00)	0.12	0.59	0.09
**Sex (M:F)**	16:10	50:18	17:12	33:6	29:10	21:8	0.38	0.03	1.00
**HBeAg positive, n(%)**	/	40(58.82)	9(31.03)	30(76.92)	/	/	/	<0.001	/
**HBsAg, Log_10_ (U/L)**	/	3.74 ± 0.74	3.55 ± 0.80	3.75 ± 0.68	4.03 ± 0.53	3.21 ± 0.53	/	0.27	<0.001
**HBV DNA, Log_10_ (IU/ml)**	/	4.15(2.90,7.79)	2.50(1.70,7.33)	6.47(4.68,7.82)	7.73(6.36,7.90)	2.50(1.70,4.15)	/	0.001	<0.001
**ALT,IU/L**	26.00(18.00,26.50)	37.00(19.50,237.50)	23.00(15.50,33.50)	191.50(120.25,365.00	152.50(64.75,349.00	149.00(33.00,239.40	<0.001	<0.001	0.001
**AST,IU/L**	18.00(16.00,26.00)	31.00(17.50,88.50)	18.00(16.50,22.00)	80.50(48.25,163.25)	73.00(36.50,161.75)	44.00(21.00,101.60)	<0.001	<0.001	<0.001
**WBC,×10^9^/L**	4.98(4.98,5.29)	5.28 ± 1.19	5.71 ± 1.06	5.30 ± 1.36	5.185.30 ± 1.28	5.84 ± 1.15	0.06	0.25	0.05

HCs, healthy controls; CHB, chronic hepatitis B; ALT, Alanine aminotransferase; AST, aspartateaminotransferase; HBeAg, hepatitis B e antigen; HBsAg, hepatitis B surface antigen; WBC, white blood cells; nor., normal; abnor., abnormal; pos., positive; neg., negative; P*, CHB Vs HC; P^#^, ALT nor. Vs ALT abnor. ALT; P^$^, HBeAg pos. Vs BeAg neg.

### Serum IL-35 Levels Were Correlated With Liver Flare and Viral Replication

**Figure 1:**
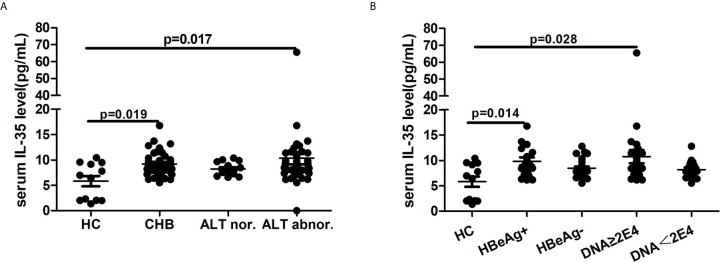
Serum IL-35 level in patients with chronic hepatitis B. 68 CHB patients were enrolled in this assay. Non-parametric Mann-Whitney U and Non-parametric Kruskal-Wallis ANOVA test were used to evaluate the differences between two groups and among groups more than two, respectively. P value<0.05 was considered statistically significant. **(A)** Serum IL-35 level in patients with different degree liver inflammation; **(B)** Serum IL-35 level in patients with different viral replication level. Data are shown as mean ± s.e.m.

To determine the role of IL-35 in persistent HBV infection, we first determined the serum IL-35 concentration in 68 patients with persistent HBV infection. Compared with HCs, CHB patients had significantly increased serum IL-35 levels. Stratified analysis found that the serum IL-35 levels were significantly increased in 39 patients with liver flare (**Figure 1A**); patients with active viral replication, including 39 HBeAg+ CHB patients; and 49 patients with viral load more than 2E4 IU/mL (**Figures 1A, B**).

### IL-35+B Cells Were Enriched During Persistent HBV Infection and Correlated With Liver Injury

**Figure 2:**
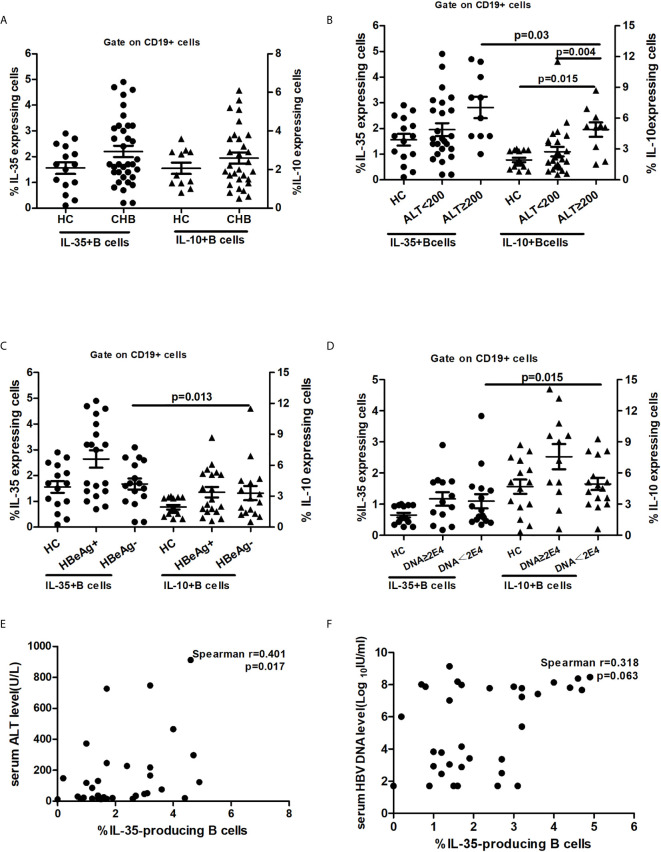
IL-35-producing B cells in patients with chronic hepatitis B. 35 CHB patients (25 of them with ALT ≤200, 17 of them with HBeAg positive and 13 of them with HBV DNA≥2E4) and 15 HCs were enrolled in this assay. Difference between two groups and among groups more than two was test by Non-parametric Mann-Whitney U test and Non-parametric Kruskal-Wallis ANOVA test, respectively. Statistical correlation between variables was calculated by the Spearman rank correlation analysis. P value<0.05 was considered statistically significant. **(A)** IL-35-producing B cell was enriched and in parallel with IL-10-producing B cells in patients with chronic hepatitis B; **(B)** Patients with chronic HBV infection had high frequency of IL-35+B and IL-10-producing B cells cells in the peripheral blood; **(C)** Frequency of IL-35-producing B cells and IL-10-producing B cells in patients with different HBeAg status; **(D)** Frequency of IL-35-producing B cells and IL-10-producing B cells in patients with different viral load; **(E)** IL-35-producing B cells were moderately correlated with ALT level; **(F)** IL-35-producing B cells were mildly correlated with serum viral load.

Breg response was defined by functional cytokines (23), and many researches have demonstrated that IL-35+B cells play a suppressive regulatory role in autoimmune and infectious diseases (22–24). However, the role of IL-35+B cells in chronic HBV infection is still unclear. To evaluate the role of circulating mononuclear cells in IL-35 secretion in persistent HBV infection, we quantified the frequency of IL-35+B cells in total B cells in 35 patients with chronic HBV infection. Compared with that in HCs, the frequency of both IL-35+B cells and IL-10-secreting B (B10) cells in total B cells in patients with chronic HBV infection was not increased significantly. (**Figure 2A**).

To analyze the relationship between IL-35+B cells and liver inflammation degree and viral replication level, we divided the treatment-naïve patients into two groups according to the ALT levels: patients with high ALT levels (ALT ≥ 200 U/L, n=25) and patients with low ALT levels (ALT < 200 U/L, n=10). Then, we also divided the patients into two groups according to HBeAg status and HBV DNA levels: patients with high virus replication (HBeAg positive, n=19; or HBV DNA ≥ 2E4 IU/mL, n=17) and patients with low virus replication (HBeAg negative, n=16 or HBV DNA < 2E4 IU/mL, n=18). Compared with HCs and patients with low ALT levels, patients with high ALT levels had the highest frequency of IL-35+B and B10 cells (**Figure 2B**). Among patients with different HBeAg status or viral loads and HCs, no significant difference was found in the frequency of IL-35+B cells (**Figures 2C, D**) in the peripheral blood total B cells. However, in patients with low viral replication, the frequency of B10 cells was higher than that of IL-35+B cells (**Figures 2C, D**). According to Spearman correlation analysis, a moderate correlation was observed between the frequency of IL-35+B cells and ALT levels (**Figure 2E**), but only mild correlation was found between the frequency of IL-35+B cells and HBV DNA levels (**Figure 2F**). The frequency of B10 cells was only moderately correlated with serum ALT levels in these patients (**Figure S3**).

### Increased Frequency of IL-35+B Cells Was Correlated With Classical Breg Subsets in Patients With Chronic HBV Infection

**Figure 3:**
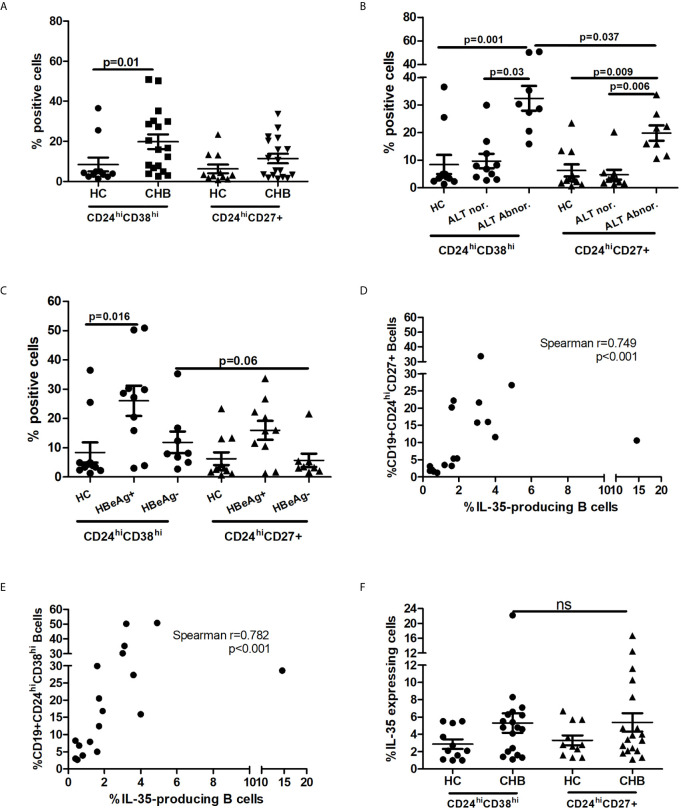
Breg subsets in CHB patients and healthy controls (HCs). Two human classical Breg subsets was gated on CD19+ cell. **(A)** Frequency of CD19+CD24^hi^CD38^hi^ or CD19+ CD24^hi^CD27+ Breg subsets in total CHB patients and HCs; **(B)** Frequency of CD19+CD24^hi^CD38^hi^ or CD19+ CD24^hi^CD27+ Breg subset in CHB patients according to different liver inflammation; **(C)** Frequency of CD19+CD24^hi^CD38^hi^ or CD19+ CD24^hi^CD27+ Breg subsets in CHB patients according to different virus replication level; **(D)** The frequency of IL-35-producing B cells was strongly correlated with the frequency of CD19+ CD24^hi^CD27+ Breg subset; **(E)** The frequency of IL-35-producing B cells was strongly correlated with the frequency of CD19+CD24^hi^CD38^hi^ Breg subset; **(F)** Higher frequency of IL-35-positive Breg subset in patients with chronic HBV infection. Difference between two groups and among groups more than two was test by Non-parametric Mann-Whitney U test and Non-parametric Kruskal-Wallis ANOVA test, respectively. Statistical correlation between variables was calculated by the Spearman rank correlation analysis. P value<0.05 was considered statistically significant. ns, no significant difference.

To quantify the frequency of various Breg subsets, the PBMCs of 33 CHB patients (17 HBeAg-positive patients and 16 patients in the abnormal ALT group) and 11 HCs were isolated and stimulated with CpG plus CD40L and LPS *in vitro* for 48 h, which was proved to potently induce Breg differentiation (22, 25). Then, the frequency of classical human Breg subsets CD19+CD24^hi^CD38^hi^ and CD19+CD24^hi^CD27+ was quantified using flow cytometry. Compared with HCs, the frequency of CD19+CD24^hi^CD38^hi^ (19.76% ± 3.64% vs. 8.43% ± 3.46%) and CD19+CD24^hi^CD27+ (11.42% ± 2.37% vs. 6.28% ± 2.18%) increased in patients with CHB (**Figure 3A**). Further stratified analysis of the frequency of Breg subsets in patients according to HBV replication and liver inflammation degree showed that patients with active liver inflammation (ALT ≥ 2*ULN) and virus replication (HBeAg positive) had higher frequency of Breg subsets, and in these patients, the Breg subset was dominated by CD19+CD24^hi^CD38^hi^ (**Figures 3B, C**).

As IL-35 is one of the markers of phenotypic changes in B cells, we analyzed the relationship of the frequency of IL-35+B cells with human classical Breg subsets during persistent HBV infection. As shown in **Figures 3D, E**, the frequency of IL-35+B cells was strongly correlated with that of two classical human Breg subsets during persistent HBV infection (Spearman r = 0.749 and 0.782, P < 0.001). Furthermore, we found that patients with persistent HBV infection had higher frequency of IL-35-positive Breg subsets compared with HCs, but no difference was observed between the two classical Bregs (**Figure 3F**).

### Correlation of IL-35+B Cells and Th1/Th2 Balance in CHB Patients

The main function of Bregs is to regulate the differentiation of Th cells and exert suppressive function on T cell proliferation through their effector factors, such as IL-10, IL-35, and TGF-β1. To evaluate the function of IL-35+B cells during persistent HBV infection, the frequency of IL-35+B cells and homologous effector T cell subsets was measured, and the correlation of IL-35+B cells with various T cell subtypes in CHB patients was analyzed using Spearman’s rank correlation analysis. We found that the frequency of IL-35+B cells was negatively correlated with that of IFN-γ-producing CD4+ and CD8+ cells, but positively correlated with that of IL-4-producing T cells (**Table 2**). However, a weaker correlation was found between serum IL-35 levels and different T cell effector cytokines (**Table S2**).

**Table 2:** Correlation between frequency of IL-35-producting B cells or Bregs with various T cell subsets.

Characteristics		Chronic HBV infection	
Condition (1)	Condition (2)	Coefficient	P value
**%IL-35-producing B cells**	**%IL-4+CD4+**	0.351	0.093
	**%IFN-γ+CD4+**	-0.073	0.693
	**%IL-17A+CD4+**	0.062	0.772
	**%IL-4+CD8+**	0.260	0.151
	**%IFN-γ+CD8+**	-0.399	0.024
**%CD19+CD24^hi^CD38^hi^**	**%IL-4+CD4+**	.298	0.230
	**%IFN-γ+CD4+**	0.104	0.681
	**%IL-17A+CD4+**	0.067	0.790
	**%IL-4+CD8+**	0.516	0.028
	**%IFN-γ+CD8+**	0.286	0.250
**%CD19+CD24^hi^CD27+**	**%IL-4+CD4+**	0.364	0.137
	**%IFN-γ+CD4+**	0.042	0.868
	**%IL-17A+CD4+**	0.086	0.734
	**%IL-4+CD8+**	0.628	0.005
	**%IFN-γ+CD8+**	0.276	0.268

In bold: %IL-35-producing B cells: Percent of IL-35-producing B cells on total B cells; %CD19+CD24hiCD38hi: percent of CD24hiCD38hi B cells on total B cells; % CD19+CD24hiCD27+: percent of CD24hiCD27+B cells on total B cells; %IL-4+CD4+: percent of IL-4 positive CD4+T cells on total CD4+T cells; %IFN-g+CD4+: percent of IFN-g positive CD4+T cells on total CD4+T cells; %IL-17A+CD4+: percent of IL-17A positive CD4+T cells on total CD4+T cells; %IL-4+CD8+: percent of IL-4 positive CD8+T cells on total CD8+T cells; %IFN-g+CD8+: percent of IFN-g positive CD8+T cells on total CD8+T cells.

### Suppression Effect on Effector Cells

**Figure 4:**
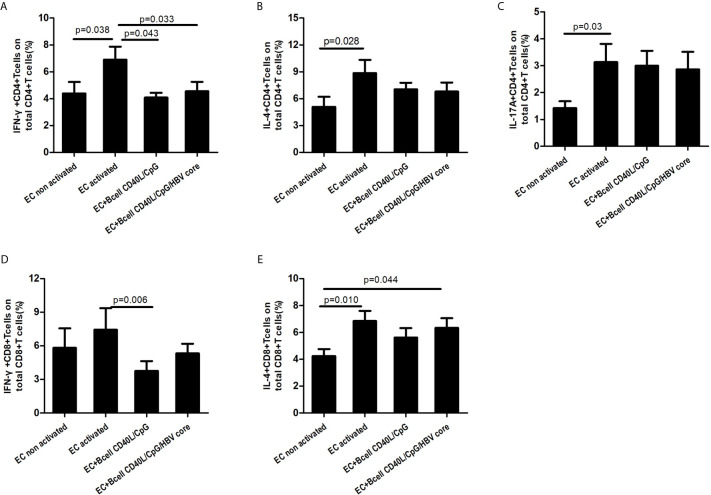
Suppression effect on effector cells (ECs). B cells were isolated from PBMC using CD19+ microbead and stimulated with CD40L/CpG/LPS with or without HBVcore peptide. Stimulated B cells were then co-cultured with autologous CD19-depleted PBMCs at the ratio of 2:1 in anti-CD3/CD28 coated plate for 48hours. As a control, CD19-depleted PBMCs were also cultured alone without B cells (CD19-depleted PBMCs non-activated and CD19-depleted PBMCs activated). **(A)** Suppression effect on IFN-ɤ-producing CD4+T cells; **(B)** Suppression on IL-4-producing CD4+T cells; **(C)** Suppression effect on IL-17A-producing CD4+T cells; **(D)** Suppression on IFN-ɤ-producing CD8+T cells; **(E)** Suppression on IL-4-producing CD8+T cells. Non-parametric Kruskal-Wallis ANOVA test and Tukey’s Multiple Comparison test was used to analysis the data. P value<;0.05 was considered statistically significant.

To determine the suppressive effect of Bregs on T cells, B cells isolated from PBMCs using anti-CD19 microbeads were stimulated with CpG/CD40L/LPS with or without HBVcore peptide for two days. Then, autologous CD19-depleted PBMCs were co-cultured with HBV-exposed B cells or stimulated B cells and subsequently activated with anti-CD3/anti-CD28 for another 48 h. Because of the importance of Th1/Th2 imbalance in CHB disease progression, we aimed to determine the regulatory effect of Bregs on IL-4- and IFN-γ-producing T cells. As shown in **Figures 4A–E**, ECs were stimulated significantly *in vitro* by anti-CD3/CD28, and Bregs significantly decreased the frequency of IFN-γ-producing CD4+ and CD8+ T cells (**Figures 4A, D**). However, the frequency of IL-4-producing T cells and IL-17-producing T cells were not significantly affected by Bregs (**Figures 4B, C, E**).

### Bregs Play Immunoregulatory Roles by Secreting IL-35 During Persistent HBV Infection

**Figure 5:**
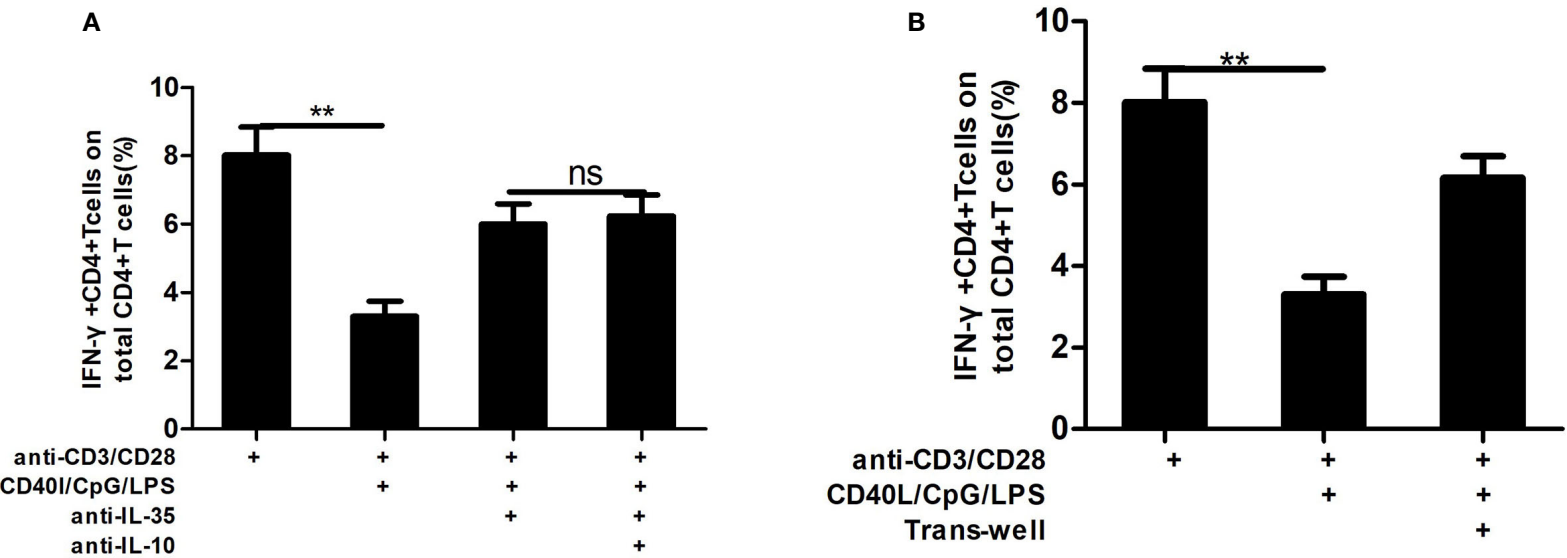
IL-35 neutralizing antibody blocking study and trans-well assay. To explore whether IL-35 worked alone or in collaboration with IL-10 in the process of Bregs regulating on T cell function, neutralizing antibody against IL-35 (1μg/ml, R&D Systems) alone or with neutralizing antibody against IL-10 (5 μg/ml, invitrogen) was added in the co-culture system of anti-CD3/anti-CD28 activated CD19-depleted PBMCs and Bregs. **(A)** Neutralizing antibody against IL-35 can block suppression function of Bregs on CD4+T cytokine secreting function; **(B)** Bregs suppressed CD4+T cell cytokine secreting depending on cell-to-cell contact. Non-parametric Kruskal-Wallis ANOVA test and Tukey’s Multiple Comparison test was used to analysis the data. P value<0.05 was considered statistically significant. **p < 0.01. ns, no significant difference.

Das et al. (17) had shown that Bregs exert their immunoregulatory role by secreting IL-10 during persistent HBV infection. We aimed to determine whether the IL-35 pathway is also involved in Bregs’ suppressive function during persistent HBV infection. As shown in **Figure 5A**, IL-35 neutralizing antibody could significantly reverse the suppressive effect of Bregs on EC cytokine production, but no synergistic function of IL-35 and IL-10 neutralizing antibody was found. Furthermore, we found that the suppressive function of Bregs on ECs was significantly decreased in the trans-well co-culture system (**Figure 5B**).

## Discussion

Bregs, especially B10 cells, are involved in the process of chronic HBV infection, such as CHB (17, 18, 26, 27), HBV-related liver cirrhosis (28), and chronic HBV infection-related renal injury (29) *via* an IL-10-dependent manner. These findings have prompted studies into the mechanism of B-cell-mediated immune regulation, particularly the suppressive cytokines secreted by B cells. During the process of chronic HBV infection, changes in serum IL-35 levels were observed compared with HCs (21, 30). Shi YY et. al (30). found that in the process of persistent HBV infection, the serum IL-35 levels increased gradually along with disease progression and were positively correlated with the liver inflammation degree. However, Cheng et al. (21) found that serum IL-35 levels were significantly decreased in patients with chronic HBV infection and negatively correlated with serum HBV DNA load and ALT levels. In our previous study (31) and the current research, we confirmed that the serum IL-35 levels in treatment-naïve chronic HBV patients were significantly increased compared with those in HCs, and patients with higher ALT levels (>200 or 300 IU/L) or HBeAg-positive patients had higher serum IL-35 levels. Our findings are consistent with those of Shi et al., suggesting that IL-35 exerts an anti-inflammatory effect during persistent HBV infection. However, the difference between the findings of Cheng et al. (21) and ours suggested that more research is needed to determine the expression and role of IL-35 at different stages of HBV infection.

The role of Bregs in chronic HBV infection was identified by Das et al. (17) in 2012. Since then, several studies have investigated the role of Bregs in chronic HBV infection and HBV infection-related mergers (18, 26, 28, 29). However, these studies only focused on the role of B10 cells. In the present study, we confirmed for the first time that IL-35+B cells were enriched and in parallel with B10 cells in patients with persistent HBV infection. Similar to B10 cells, the frequency of IL-35+B cells was associated with liver inflammation and HBV replication level. These findings suggested that in the process of persistent HBV infection, B cells may exert immune regulation through different pathways. Furthermore, we also confirmed that two typical human Breg subsets (CD19+CD24^hi^CD38^hi^ and CD19+CD24^hi^CD27+) were enriched and strongly correlated with the frequency of IL-35+B cells. These results further demonstrated that IL-35+B cells mainly belong to Breg subsets.

Previous studies had demonstrated that Bregs are enriched and exert a regulatory role on multiple immune ECs including T cells in CHB patients (17, 27, 32). These studies showed that Bregs play a regulatory role on HBV-specific T cell responses in an IL-10-dependent manner by inhibiting TNF-α and IFN-γ production and enhancing Treg function. In the present study, we confirmed that Bregs can inhibit IFN-γ production but has little effect on IL-4 production. The inhibitory effect of Bregs on IFN-γ production could be reversed by IL-35 neutralizing antibody *in vitro*, and the inhibitory effect was dependent on cell-to-cell contact. These results suggest that during HBV infection, Bregs participate in the T cell imbalance regulation process through an IL-35-dependent manner. To our knowledge, this is the first time to determine that that during persistent HBV infection, Bregs exert immune regulation through a channel other than IL-10-dependent pathway. However, in other chronic virus infections, such as HIV infection, Lopez-Abente et al. (33) had confirmed that HIV-1 could induce B cell toward the Breg phenotype and express IL-10, TGF-β, and EBI3 or IL-12(p35) mRNA.

In conclusion, during persistent HBV infection, serum IL-35+B cells exited and was enriched in CD19+CD24^hi^CD38^hi^ B cell subset. Bregs dysregulated T cell function through an IL-35-dependent mechanism, which depended on cell-to-cell contact.

## Data Availability Statement

The original contributions presented in the study are included in the article/**Supplementary Material**. Further inquiries can be directed to the corresponding author.

## Ethics Statement

The studies involving human participants were reviewed and approved by Clinical Ethics Committees of Shanghai Changhai Hospital. The patients/participants provided their written informed consent to participate in this study.

## Author Contributions

YYL, YL, TZ, and MJ were involved in recruiting patients, collecting data, and performing a series of *in vitro* experiments. ZFT and GST assisted the intracellular labeling flow cytometric analysis, and data analysis. YYL, MJ and XSL involved in the study concept and design, and XSL obtained funding. XSL performed the data analysis and drafted the manuscript. All authors contributed to the article and approved the submitted version.

## Funding

This study was supported by Natural Science Foundation of Shanghai (16ZR1400400; 20ZR1456900) and Wu Jieping Medical Foundation (LSWJPMF-102-17001). Fund units are not involved in the design, analysis and conduct of this study.

## Conflict of Interest

The authors declare that the research was conducted in the absence of any commercial or financial relationships that could be construed as a potential conflict of interest.
