# Supplementary material for: Regulatory B Cells Dysregulated T Cell Function in an IL-35-Dependent Way in Patients With Chronic Hepatitis B
Source: Front Immunol. 2021 Apr 12;12:653198. doi: 10.3389/fimmu.2021.653198 (PMC8072152; doi:10.3389/fimmu.2021.653198)
Supplement: Supplementary file 1 [file DataSheet_1.docx]

**Table S1: Antibodies and fluorochromes used in this work**

| **PBMC** | **Intracelular labeling** | |
| --- | --- | --- |
| **B cells and Breg panel** | **Th1/Th2/Th17 panel** | **IL-10/IL-35 panel** |
| CD3-V500-A | CD3-APC-Cy7-A | CD3-V500-A |
| CD19-APC-Cy7-A | CD4-FITC-A | CD19-APC-Cy7-A |
| CD38-PE-A | CD8-PerCP-Cy5.5-A | CD38-PE-A |
| CD27-PE-Cy7-A | IL-17-APC-A | CD27-PE-Cy7-A |
| CD24-FITC-A | IFN-g PE-Cy7-A | IL-10-V450-A |
| Ghost Dye Red 780 | IL-4-PE-A | eBi3-PerCP-Cy5.5-A |
|  |  | IL-12/IL-35p35 APC-A |

**Table S2: Correlation between serum IL-35 level and different T cell effector cytokines**

| **Characteristics and conditions** | | HCs | | CHB | |
| --- | --- | --- | --- | --- | --- |
| **Condition (1)** | **Condition (2)** | **Coefficient** | **P value** | **Coefficient** | **P value** |
| **IL-35** | **IFN-γ** | **-0.129** | **0.674** | **0.069** | **0.747** |
|  | **IL-17A** | **0.296** | **0.350** | **0.139** | **0.517** |
|  | **IL-2** | **0.056** | **0.862** | **0.151** | **0.502** |
|  | **IL-21** | **0.252** | **0.430** | **-0.194** | **0.364** |
|  | **IL-4** | **-0.114** | **0.738** | **0.024** | **0.912** |

Note: HCs: healthy controls; CHB: chronic HBV infection; IL-35: interleukine-35; IFN-γ：interferon-γ; IL-17A: interleukine-17A; IL-2: interleukine-2; IL-21: interleukine-21; IL-4: interleukine-4.

**
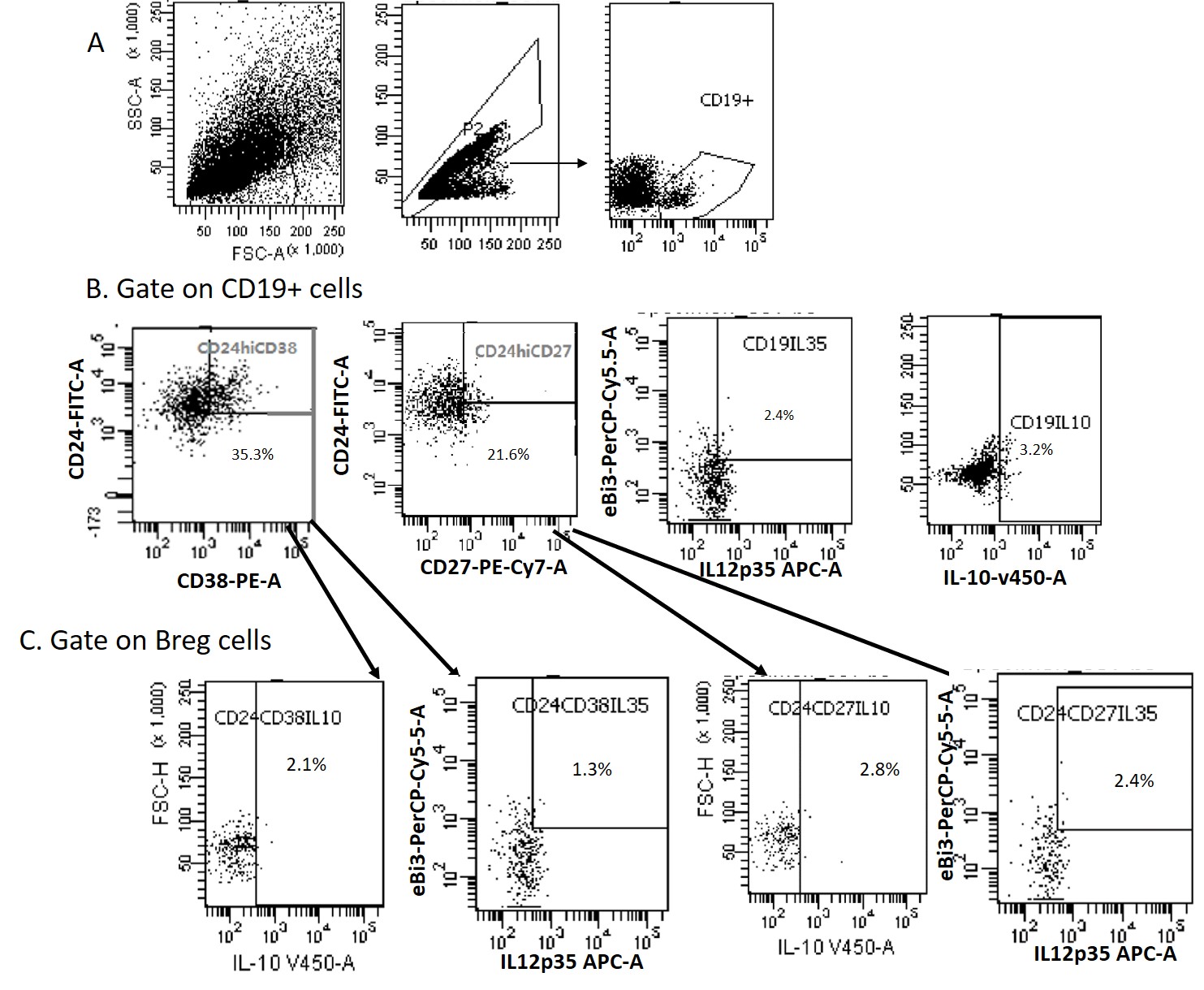
**

**Figure S1: Gating strategies and example for the flow data of B cell subsets.**

**
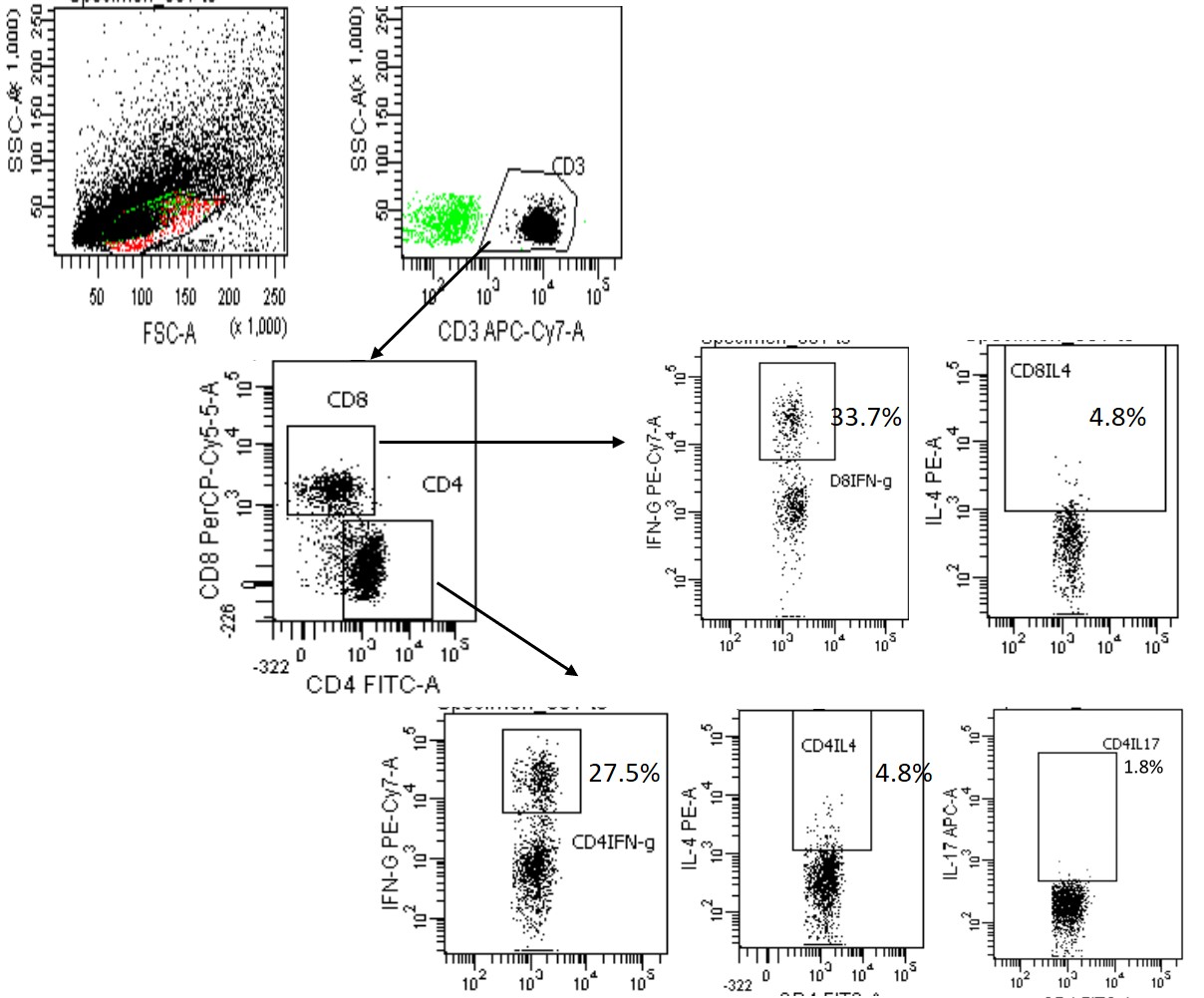
**

**Figure S2: Gating strategies and example for the flow data of T cell subsets.**

**
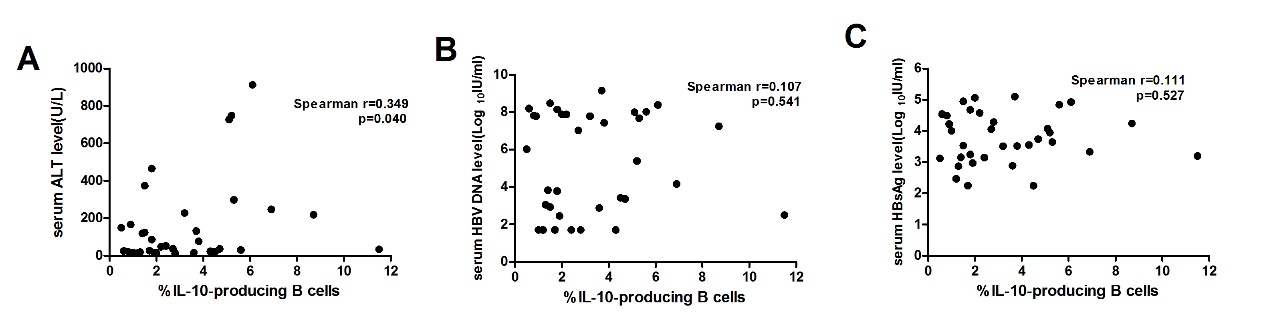
**

**Figure S3: Correlation of IL-10-producing B (B10) cells and liver inflammation and viral replication.** A: The frequency of B10 cells was positively correlated with liver inflammation; B: Correlation of the frequency of B10 cells and serum viral load; C: Correlation of the frequency of B10 cells and serum HBeAg level. Statistical correlation between variables was calculated by the Spearman rank correlation analysis. P value﹤0.05 was considered statistically significant.
